# Unilateral laminotomy with bilateral spinal canal decompression: systematic review of outcomes and complications

**DOI:** 10.1186/s12891-023-07033-1

**Published:** 2023-11-21

**Authors:** Nizar Algarni, Mohamed Al-Amoodi, Yousef Marwan, Rakan Bokhari, Abdullah Addar, Abdullah Alshammari, Abdulrahman Alaseem, Waleed Albishi, Ibrahim Alshaygy, Fahad Alabdullatif

**Affiliations:** 1 Department of Orthopedic Surgery, College of Medicine, King Saud University, Riyadh, Saudi Arabia; 2 Department of Orthopedics, University of British Columbia, Vancouver, BC Canada; 3 Department of Surgery, Faculty of Medicine, Health Sciences Center, Kuwait University, Kuwait City, Kuwait; 4 Division of Neurosurgery, Faculty of Medicine, King Abdulaziz University, Jeddah, Saudi Arabia; 5 Department of Orthopedic Surgery, McGill University Health Centre, Montreal, QC Canada

**Keywords:** Laminotomy, Spinal decompression, Spine, Spinal stenosis

## Abstract

**Background:**

Unilateral laminotomy with bilateral spinal canal decompression has gained popularity recently.

**Aim:**

To systematically review the literature of unilateral laminotomy with bilateral spinal canal decompression for lumbar spinal stenosis (LSS) aiming to assess outcomes and complications of the different techniques described in literature.

**Methods:**

On August 7, 2022, Pubmed and EMBASE were searched by 2 reviewers independently, and all the relevant studies published up to date were considered based on predetermined inclusion and exclusion criteria. The subject headings “unilateral laminotomy”, “bilateral decompression” and their related key terms were used. The Preferred Reporting Item for Systematic Reviews and Meta-Analyses statement was used to screen the articles.

**Results:**

A total of seven studies including 371 patients were included. The mean age of the patients was 69.0 years (range: 55–83 years). The follow up duration ranged from 1 to 3 years. Rate of postoperative pain and functional improvement was favorable based on VAS, JOA, JOABPEQ, RMDW, ODI and SF-36, for example improved from a range of 4.2–7.5 preoperatively on the VAS score to a range of 1.4–3.0 postoperatively at the final follow up. Insufficient decompression was noted in 3% of the reported cases. The overall complication rate was reported at 18–20%, with dural tear at 3.6–9% and hematoma at 0–4%.

**Conclusion:**

Unilateral laminotomy with bilateral decompression has favorable short- and mid-term pain and functional outcomes with low recurrence and complication rates. This, however, needs to be further confirmed in larger, long-term follow-up, prospective, comparative studies between open, and minimally invasive techniques.

## Introduction

Lumbar spinal stenosis (LSS) refers to narrowing of the central canal and occasionally the neural foramina within the lumbar spine, which predominantly manifest as a degenerative condition affecting the elderly population [1]. Compression arises from disc protrusion, facet or ligamentum flavum hypertrophy, or osteophytes [1]. The condition can be associated with translation of two adjacent vertebrae introducing another source of compression, that is known as spondylolisthesis [1].

LSS is a common source of back pain affecting approximately 100 million people worldwide [2]. But in fact, it has been shown that around 20% of elderly people have some form of lumbar stenosis, of which only about 20% are symptomatic [3]. While asymptomatic patients and the majority of those who are symptomatic are treated non-operatively, a large number do undergo surgery [1]. A national survey conducted in the US reported that about 350,000 individuals over 45 underwent laminectomies alone and about the same number underwent fusion mostly for the treatment of LSS [4].

Besides back pain, patients also complain of buttocks pain, radiculopathy, which could be unilateral or bilateral, paresthesia or weaknesses [1, 5–7]. As it progresses, proprioceptive deficits could lead to gait instability [5–7].

Clinicians rely on a good history, physical examination, and imaging to make the diagnosis [8]. Patients typically complain of lower back pain, with radiculopathy, that is typically relieved with lumbar flexion (e.g., leaning on a shopping cart) or sitting but can’t tolerate prolonged lumbar extension [1, 5, 6, 8]. Sensory and motor deficits can also be observed along the L3-S1 distribution with sensitivity of 50% [8]. There is no single diagnostic tool for this condition, and sometimes a computed tomography (CT) scan or magnetic resonance imaging (MRI) can be helpful to determine the cross-sectional area of the spinal canal [3, 5, 9]. There is no consensus on the spinal canal cross-sectional area cutoff for diagnosis of LSS, however, an area below 191 mm^2^ has a sensitivity and specificity of 93% and 45%, respectively [9]. The most common level of LSS is L4-5 followed by L3-4 in 92% and 66%, respectively. Plain radiographs are also useful to assess dynamic stability, as 5 mm spondylolisthesis can occur in up to 34% of individuals with lumbar stenosis [10].

The simplest form of non-operative management is activity modification by favoring flexion position, such as cycling [11]. Many studies have looked into non-steroidal anti-inflammatory drugs (NSAIDs), though it is unclear how effective they are in the management of LSS [11]. However, the addition of neuropathic analgesics such as gabapentin have been shown to provide multi-modal benefits with reduction of 2.1 points on the visual analogue scale [12]. Epidural steroid injections have also been studied; similar to NSAIDs, their benefits have been challenged by a recent randomized trial that showed that there may be a component of placebo that provides pain relief beyond the effective duration of short acting analgesics [13].

In patients for whom non-operative management has failed or the symptoms are severe enough to warrant an intervention, it may be necessary to undergo a decompressive surgery. With respect to the decompression itself, conventional posterior open laminectomy is associated with considerable trauma to the paraspinal muscles, which in turn could result in pain [14]. One proposed solution to this problem is performing a unilateral window to decompress both sides of the spine [15]. Another emerging intervention is endoscopic ULBD as described by Hyeun-Sung Kim et al [16] however, the steep learning curve considered one of the major disadvantage of this technique. Spetzger et al. have provided a technique that can successfully achieve adequate decompression using a unilateral window [15]. The technique has gained popularity recently, because it reduces trauma to the muscles, as well as the interspinous and supraspinous ligaments which in theory would result in better outcomes [16].

In this study, we aim to systematically review the literature of unilateral laminotomy with bilateral decompression to assess the outcomes and complications of this recently popularized technique. We hypothesize that overall outcomes are favorable with regards to functional outcomes, recurrence rate and complications.

## Materials and methods

This systematic review was conducted in accordance with the preferred reporting items for systematic reviews and meta-analyses (PRISMA) guidelines [17].

### Search strategy

PubMed and EMBASE databases were searched independently by two authors for relevant articles until August 7, 2022. The search was limited to English language only. The subject headings “unilateral laminotomy”, “bilateral decompression” and their related key terms were used. The articles were screened based on the Preferred Reporting Item for Systematic Reviews and Meta-Analyses statement.

### Inclusion and exclusion criteria

The following inclusion criteria were used in our systematic review: (1) clinical studies; (2) all levels of evidence; (3) unilateral laminotomy with bilateral decompression for lumbar spinal stenosis; and (4) no restriction to date of publication. Studies were excluded if they met any of the following criteria: (1) non-English articles; (2) articles published in abstract form only; (3) review articles; (4) technique articles; (5) decompression with fusion articles; (6) endoscopic technique articles and (7) cadaveric or animal studies. Disagreements were sorted by group discussion with the authors.

### Data collection/extraction

Independent screening of the titles and abstracts of the included studies were carried out by the same authors. Articles were included in the full-text review stage if any of the authors believed it should, and further filtered during this stage. The data was then retrieved from the included studies and entered in Microsoft Excel 2013 (Microsoft, Redmond, WA, United States) independently by the same authors. The information was categorized into basic background/clinical data (e.g., title, authors, year of publication, country of publication, sample size, sex, age, risk factor and preoperative assessment), surgical technique (e.g., tools, techniques, and other surgical details) and postoperative outcomes and complications (e.g., follow-up duration, recurrence, complications, pain and functional scores). The primary outcome of this review was pain improvement, while recurrence rate, functional score changes, and complications were the secondary outcomes.

### Statistical analysis

All statistical analyses were performed using R version 3.5.1 (R Foundation for Statistical Computing, Vienna, Austria). Since numerical data were often missing important values such as standard deviation, a meta-analysis could not be performed. Therefore, descriptive analysis and weighted means were per- formed on the numerical data.

## Results

**Fig. 1:**
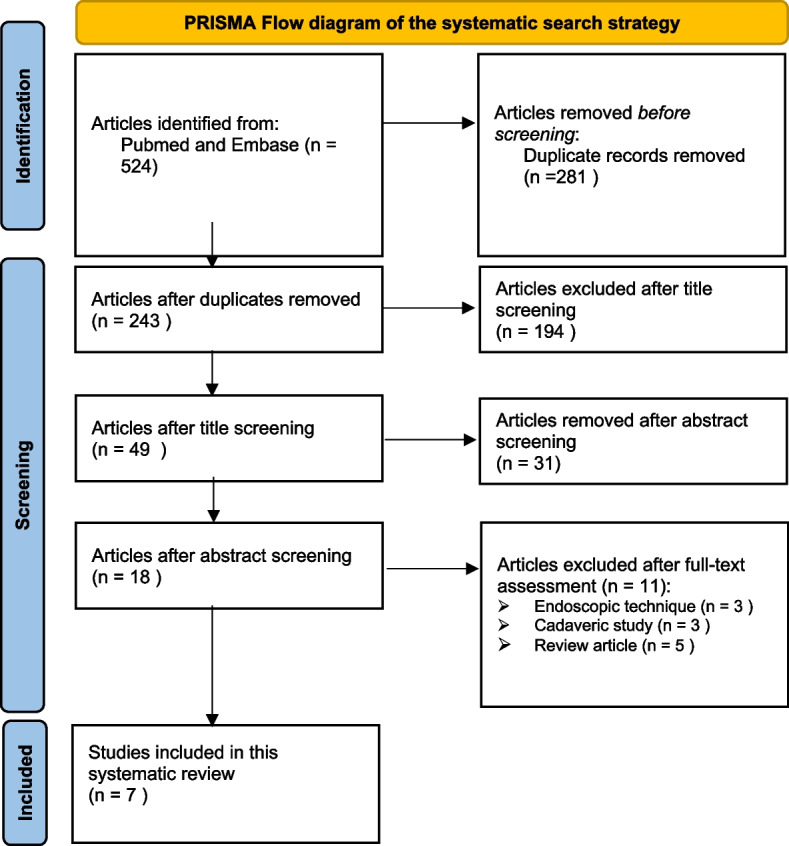
PRISMA Flow diagram of the systematic search strategy

The databases, Pubmed and EMBASE, initially revealed a total of 524 studies, reduced to 243 after duplicates were removed (Fig. 1). A total of 194, 31 and 11 studies were excluded after title, abstract and full-text review, respectively. Therefore, seven studies were included for the final analysis [18–24]. These articles were published from North America, Asia and Europe. Additional screening of the references of the seven articles did not reveal more relevant studies that met the inclusion criteria. The 2 reviewers had no disagreements throughout the stages of the systematic review.

Table 1 summarizes the background and clinical information of patients with LSS who underwent unilateral laminotomy with bilateral spinal canal decompression. Three hundred seventy-one patients underwent unilateral laminotomy with bilateral spinal canal decompression, of whom 189 (50.9%) were males. The mean age of the patients was 69.0 ± 9.0 years (range: 55–83 years). X-rays (XR), CT, CT myelogram and MRI were used to help establish the diagnosis of LSS. Six out of the seven studies used MRI, while three studies used CT scans. The Visual Analogue Scale (VAS) score was used to assess the severity of pain in six out of seven studies (243 patients; 65.5%); however, the scaling system was inconsistent amongst the studies, including the use of a 0–10, 1–10, or a 0–100 range. Other scoring systems used included: the Japanese Orthopedic Association (JOA) score which was used in a single study (50 patients; 13.5%), the Japanese Orthopedic Association Back Pain Evaluation Questionnaire (JOABPEQ) score which was used in a single study (50 patients; 13.5%), the Roland–Morris Disability Questionnaire (RMDQ) score which was used in a single study (25 patients; 6.7%), the Oswestry Disability Index (ODI) score which was used in five studies (193 patients; 52.0%) and the Short Form health survey (SF-36) score which was used in a single study (25 patients; 6.7%).

**Table 1:** Background and clinical information of patients with LSS who underwent unilateral laminotomy with bilateral spinal canal decompression

Ref	Sample size	Sex	Age	Risk factor	Diagnostic investigation	Preop. VAS	Preop. JOA	Preop. JOABPEQ	Preop. RMDW	Preop. ODI	Preop. SF-36	Preop. Spinal Stenosis Measure	Preop. EQ-5D-3L
			(years mean ± S.D.)			(mean ± S.D.)	(mean ± S.D.)	(mean ± S.D.)	(mean ± S.D.)	(mean ± S.D.)	(mean ± S.D.)	(mean ± S.D.)	(mean ± S.D.)
Arai et al. [18], 2014	50	34 M; 16 F	69.5 ± 8.7	8 with spondylolisthesis	CT looking at lumbar spondylosis	6.41 ± 2.81	14.5 ± 5.1	40.4 ± 38.6	-	-	-	-	-
Yang et al. [19], 2020	28	18 M; 10 F	73.0 ± 5.9	-	MRI central stenosis grade	4.68 ± 0.94 (back) 6.07 ± 0.81 (leg)	-	-	-	61.86 ± 7.32	-	-	-
Ko et al. [20], 2019	25	8 M; 17 F	68.1 ± 10.7	-	MRI showing central stenosis and flexion/extension XR for dynamic instability	4.20 ± 2.20 (back) 7.28 ± 1.22 (leg)	-	-	-	-	-	-	-
McGrath et al. [21], 2019	45	27 M; 18 F	62.0 ± 1.3	23 with spondylolisthesis	MRI showing central stenosis and flexion/extension XR for dynamic instability	7.1 ± 0.4 (back) 6.3 ± 0.5 (leg)	-	-	-	47.2 ± 3.1	-	-	-
Ulrich et al. [22], 2019	128	62 M; 66 F	72.9 ± 7.9	57 with degenerative spondylolisthesis	MRI central stenosis grade	-	-	-	-	-	-	3.1 ± 0.6 (symptoms) 2.3 ± 0.7 (function)	67.3 ± 15.1
Mobbs et al. [23], 2014	27	5 M; 22 F	72.7 ± 10.4	-	MRI or CT myelogram confirming central stenosis	7.5 ± 2.1 (leg)	-	-	-	51.4 ± 19.4	-	-	-
Knio et al. [24], 2019	68	35 M; 33 F	65.0 ± 9.2	23 with spondylolisthesis	MRI or CT with central stenosis	7.3 ± 3.0 (back) 7.5 ± 2.7 (leg)	-	-	-	51.2 ± 14.5	-	-	-

Table 2 demonstrates the surgical techniques used in the included studies. Each group of surgeons used a slightly different set of tools and techniques; however, the basic setup consisted of a microscope and high-speed drill burr. The technique used by Arai et al. [18] and Ulrich et al. [22] followed the original technique described by Spetzger et al. [15] which involved a midline approach, dissecting the paraspinal muscles down to the interlaminar space. Subsequently under microscopic view a laminotomy was performed by removing a portion of the superior and inferior laminae, a small portion of the medial facet and the ligamentum flavum at the segment exposing the dural sac [15]. The operative table and microscope were tilted towards the contralateral side, then with the help of a high-speed burr, deep cortical surface undercutting was performed on the contralateral lamina up to the contralateral lateral recess, and similarly a flavectomy was performed contralaterally. The nerve roots were effectively decompressed. The technique used by Yang et al. [19] was very similar, but a Casper was used to aid in retraction and the side dissected was the painful side. Ko et al. [19] diverted slightly from Spetzger et al. [15] by not only specifying the use of the right side for initial dissection, but also performing a flavectomy of the contralateral side only in severe cases and avoidance of undercutting the base of the spinous process. McGrath et al. [21] augmented the approach with an endoscope, the ILESSYS delta system. Their setup relies on using a Wilson frame to achieve kyphosis on a Jackson table; after confirming the level, a stab incision is made, serial dilators are introduced until the inferior lamina is palpable, a tubular retractor is then introduced to fit the endoscope, and a burr is used to remove the inferior portion of the superior lamina and the medial aspect of the ipsilateral facet exposing the ligamentum flavum, which was then resected with a Kerrison rongeurs. The burr is then used on the contralateral side until the pedicles are visualized, confirming contralateral nerve root decompression. Mobbs et al. [23] and very similarly Knio et al. [24] used an incision which was off the midline by 1 cm laterally, but incised 3 cm longitudinally; an 18 mm tubular retractor was used to create a corridor and a cautery to expose muscle, then a burr and Kerrison rongeur were used to decompress the canal and if needed the contralateral foramen.

**Table 2:** Surgical technique used for unilateral laminotomy with bilateral spinal canal decompression for LSS

Ref	Sample size	Surgical tools	Surgical technique	Other surgical notes
Arai et al. [18], 2014	50	Microscope, high-speed drill burr	The microsurgical procedure was performed as described by Spetzger et al. [15] with preservation of supraspinous and interspinous ligament	Decompression of 1 segment (25), 2 segments (16), 3 segments (9)
Yang et al. [19], 2020	28	Microscope, high-speed drill burr	4 cm incision median approach, dissection of paravertebral muscles of painful side unilaterally by a Casper and window exposure of interlaminar with Kerrison rongeur	Affected segment: L3-4 (3), L4-5 (23), L5-S1 (2)
Ko et al. [20], 2019	25	Microscope, high-speed drill burr	The microsurgical procedure was performed as described by Spetzger et al. [15] on the right side with preservation of supraspinous and interspinous ligament without undercutting the base of the spinous process and only performing flavectomy in severe hypertrophy on the contralateral side; 2/3 of cranial and 1/3 of caudal, 30–40 degrees inclination to resect contralateral hypertrophied ligamentum flavum	Affected segment: L3-4 (1), L4-5 (18), L5-S1 (6)
McGrath et al. [21], 2019	45	Microscope, high-speed drill burr, Wilson frame, tubular dilators, ILLESSYS delta endoscope	Endoscopic approach using a Wilson table is used after serial dilation to introduce a burr which is used to remove the inferior portion of the superior lamina and the medial aspect of the ipsilateral facet	Decompression of 1 segment (21), 2 segments (17), > 2 segments (7)
Ulrich et al. [22], 2019	128	Microscope, high-speed drill burr	The microsurgical procedure was performed as described by Spetzger et al. [15] with preservation of supraspinous and interspinous ligament	Decompression of 1 segment (47), 2 segments (55), > 2 segments (26)
Mobbs et al. [23], 2014	27	Microscope, high-speed drill burr, tubular dilators	Incision is slightly lateral to midline by 1 cm and 3 cm long; an 18 mm tubular retractor was used to create a corridor and a cautery to expose muscle; subsequently a burr and Kerrison rongeur were used to decompress the canal and if needed the contralateral foramen	Affected segment: L2-3 (1), L3-4 (5), L4-5 (23), L5-S1 (0)
Knio et al. [24], 2019	68	Microscope, high-speed drill burr, tubular dilators	A paramedian 2.5 cm incision is used; a tubular retractor is used to create a corridor, for subsequent decompression with Kerrison rongeur	Affected segment: L2-3 (3), L3-4 (13), L4-5 (48), L5-S1 (4)

The outcomes and complications of unilateral laminotomy with bilateral spinal canal decompression for LLS are demonstrated in In Table 3. The follow up duration of the 371 patients ranged from 1 to 3 years. Ko et al. [19] reported the best VAS score where leg pain was 1.20 at the 2 year follow up. The postoperative JOA and JOABPEQ scores reported by Arai et al. [18] improved from 14.5 and 40.4 to 25.6 and 80.2, respectively. Postoperative ODI, which was the second most reported scoring system, ranged from 12.8 to 28.75. Other reported outcomes, such as SF-12 and SF-36 showed favorable outcomes. Insufficient decompression was noted in 3% of the reported cases. The overall complication rate was reported at 18–20%, with dural tear at 3.6–9% and hematoma at 0–4%.

**Table 3:** Outcomes and complications of unilateral laminotomy with bilateral spinal canal decompression for LLS

Ref	Sample size	Follow-up duration	Postop. VAS	Postop. JOA	Postop. JOABPEQ	Postop. RMDW	Postop. ODI	Postop. SF-36	Other outcomes	Insufficient decompression	Operative time	Blood loss	Complications
			(mean ± S.D.)	(mean ± S.D.)	(mean ± S.D.)	(mean ± S.D.)	(mean ± S.D.)	(mean ± S.D.)			(mins mean ± S.D.)	(mL mean ± S.D.)	
Arai et al. [18], 2014	50	2 years	2.57 ± 2.94 (2 years)	25.6 ± 3.5 (2 year)	80.2 ± 32.8	-	-	-	Buttock and/or lower extremity numbness decreased from 70.3 ± 25.4 to 31.3 ± 35.4	2%	181 ± 64.6	114 ± 114	Hematoma (0%), Adjacent segment disorder (2%)
Yang et al. [19], 2020	28	2 weeks, 3 months, 6 months, 1 year	2.50 ± 0.75 (back, 1 year) 2.68 ± 1.09 (leg, 1 year)	-	-	-	28.75 ± 7.06	-	-	-	72.0 (68.8–74.8)	-	Complications (17.9%), Dural tear (3.6%), Urinary retention (7.1%), Delirium (7.1%)
Ko et al. [20], 2019	25	6 months, 1 year, 2 years	1.38 ± 1.19 (back, 2 years) 1.20 ± 1.15 (leg, 2 years)	-	-	4.60 ± 3.62	12.0 ± 8.17	64.0 ± 20.1 (PCS, 2 years) 70.4 ± 18.3 (MCS, 2 years)	-	-	119 ± 39.8	-	Did not report complications
McGrath et al. [21], 2019	45	2 weeks, 3 months, 1 year	4.2 ± 0.6 (back, 1 year) 3.0 ± 0.5 (leg, 1 year)	-	-	-	-	-	-	2%	154.1 ± 6.2	52 ± 11	Hematoma (4%), Dural tear (7%), Urinary retention (13%), Paresthesia (2%)
Ulrich et al. [22], 2019	128	1 year, 2 years, 3 years	-	-	-	-	-	-	-	5%	-	-	Complications (20.4%), Hematoma (1.6%), Dural tear (7.8%), Infection (1.6%), Mortality within 3 months (0%)
Mobbs et al. [23], 2014	27	3 years	1.9 ± 2.5 (leg, 3 years)	-	-	-	22.8 ± 27.7	-	SF-12 (PCS, 3 years) and SF-12 (MCS, 3 years) improved by 40.1 ± 10.8 and 50.2 ± 10, respectively	-	-	-	Dural tear (4%)
Knio et al. [24], 2019	68	1 year, 2 years	2.8 ± 2.8 (back, 2 years) 2.1 ± 2.8 (leg, 2 years)	-	-	-	20.3 ± 17.1	-	-	-	118.7 ± 28.9	-	Dural tear (9%)

## Discussion

A facet-preserving laminectomy via posterior midline incision has always been considered the gold-standard treatment for LSS that failed conservative management [1, 7, 14]. The technique has better visualization of the dura and nerve roots; thus, it has been thought to provide the best outcomes [15]. Unfortunately, it comes with certain disadvantages including stripping of musculoligamentous attachments of posterior spinal elements at the affected level [15]. This has been postulated to result in segmental instability, muscular weakness, and postoperative pain [15, 20, 22]. More recently popularized techniques which are less invasive could avoid this complication without sacrificing on effective decompression [16].

### Outcomes of ULBD

The current systematic review has explored the outcomes and complications of unilateral laminotomy with bilateral decompression for LSS in 371 patients. Regardless of the technique or equipment used to perform the decompression, the outcomes were excellent. Resolution of pain and improvement in functional scores were favorable based on VAS, JOA, JOABPEQ, RMDW, ODI and SF-36 scores at the final follow up assessment; Ko et al. [19] reported the best VAS score where leg pain was 1.20 at the 2 year follow up compared to 7.5 preoperatively. On the other hand, 2–5% had insufficient decompression [18–24], and while no major complications have been reported, minor complications such as dural tears and urinary retention were reported, the overall complication rate was 18–20% [18–24]. It should be noted that a major disadvantage that this method brings is the steep learning curve needed to perform the operation, because every study reported a small number of senior surgeons performing the cases.

Treating LSS with open laminectomy can achieve excellent outcomes with regards to VAS, and ODI scores at the 5 years follow up [23]. Furthermore, in comparison, the reoperation rate which has been well documented is predictable at about 12% at the 6 year follow up for patients with restenosis following standard laminectomy [23]. The disadvantages with this technique is not only the disruption of bony, or paraspinal structures previously discussed, but also the creation of a large dead space which makes for an ideal medium for bacterial colonization, and scar surrounding the dura [25–28]. These complications are additive to the instability and atrophy issues previously discussed, because the disease commonly involves multiple levels in the elderly population [25]. Thus, techniques involving minimally invasive approach might be preferable.

It is worth mentioning that endoscopic surgery techniques have been evolving over the past few years, including endoscopic ULBD [16, 29]. Beside the advantages of minimally invasive surgery, outside-in technique has the advantage of maintaining the deep layer of ligamentum flavum, providing neural elements shielding during bony decompression till the last stages of endoscopic decompression as described by Hyeun-Sung Kim et al [16]. However, further studies are needed to compare this evolving technique to the other available techniques.

### Limitations

A limitation to the study is that these differences have not been proven with high-quality, comparative, prospective studies. It is especially lacking with regards to long term data because symptoms worsen overtime. Also, this surgical technique requires advanced surgical skills and experience which may be variant from a surgeon to another. As such, we cannot provide new recommendations to guide clinical practice. Nonetheless, valuable information has been extracted from the available studies which should help guide further research.

## Conclusion

Unilateral laminotomy with bilateral decompression has favorable short- and mid-term outcomes with low recurrence and complication rates. This, however, needs to be further confirmed in larger, long-term follow-up, prospective, comparative studies between open, and minimally invasive techniques.

## Data Availability

The datasets used and/or analysed during the current study available from the corresponding author on reasonable request.

## References

[1] Katz JN, Zimmerman ZE, Mass H, Makhni MC (2022). Diagnosis and management of lumbar spinal stenosis: a review. JAMA.

[2] Ravindra VM, Senglaub SS, Rattani A (2018). Degenerative Lumbar Spine Disease: Estimating Global Incidence and Worldwide Volume. Glob Spine J.

[3] Boden SD, Davis DO, Dina TS, Patronas NJ, Wiesel SW (1990). Abnormal magnetic-resonance scans of the lumbar spine in asymptomatic subjects. A prospective investigation. J Bone Joint Surg Am.

[4] HCUP-US NIS Overview. Accessed August 29, 2022. https://www.hcup-us.ahrq.gov/nisoverview.jsp

[5] Genevay S, Atlas SJ (2010). Lumbar spinal stenosis. Best Pract Res Clin Rheumatol.

[6] Jensen RK, Lauridsen HH, Andresen ADK, Mieritz RM, Schiøttz-Christensen B, Vach W (2020). Diagnostic Screening for Lumbar Spinal Stenosis. Clin Epidemiol.

[7] Weinstein JN, Tosteson TD, Lurie JD (2008). Surgical versus nonsurgical therapy for lumbar spinal stenosis. N Engl J Med.

[8] Katz JN, Dalgas M, Stucki G (1995). Degenerative lumbar spinal stenosis. Diagnostic value of the history and physical examination. Arthritis Rheum.

[9] Lim YS, Mun JU, Seo MS (2017). Dural sac area is a more sensitive parameter for evaluating lumbar spinal stenosis than spinal canal area: a retrospective study. Medicine (Baltimore).

[10] Katz JN, Lipson SJ, Lew RA (1997). Lumbar laminectomy alone or with instrumented or noninstrumented arthrodesis in degenerative lumbar spinal stenosis Patient selection, costs, and surgical outcomes. Spine.

[11] Chou R, Deyo R, Friedly J (2017). Systemic Pharmacologic Therapies for Low Back Pain: A Systematic Review for an American College of Physicians Clinical Practice Guideline. Ann Intern Med.

[12] Yaksi A, Ozgönenel L, Ozgönenel B (2007). The efficiency of gabapentin therapy in patients with lumbar spinal stenosis. Spine.

[13] Manchikanti L, Cash KA, McManus CD, Pampati V, Fellows B (2012). Results of 2-year follow-up of a randomized, double-blind, controlled trial of fluoroscopic caudal epidural injections in central spinal stenosis. Pain Physician.

[14] Ghogawala Z, Dziura J, Butler WE (2016). Laminectomy plus Fusion versus Laminectomy Alone for Lumbar Spondylolisthesis. N Engl J Med.

[15] Spetzger U, Bertalanffy H, Naujokat C, von Keyserlingk DG, Gilsbach JM (1997). Unilateral laminotomy for bilateral decompression of lumbar spinal stenosis. Part I: Anatomical and surgical considerations. Acta Neurochir (Wien).

[16] Kim, Hyeun-Sung, Wu, Pang Hung & Jang, Il-Tae. Lumbar Endoscopic Unilateral Laminotomy for Bilateral Decompression Outside-In Approach: A Proctorship Guideline With 12 Steps of Effectiveness and Safety. Neurospine. 2020; 17: S99-S109. 10.14245/ns.2040078.039. 10.14245/ns.2040078.039 PMC7410378 32746523

[17] Moher D, Liberati A, Tetzlaff J, Altman DG, The PRISMA Group (2009). Preferred reporting Items for Systematic Reviews and Meta-Analyses: The PRISMA Statement. PLoS Med.

[18] Arai Y, Hirai T, Yoshii T (2014). A prospective comparative study of 2 minimally invasive decompression procedures for lumbar spinal canal stenosis: unilateral laminotomy for bilateral decompression (ULBD) versus muscle-preserving interlaminar decompression (MILD). Spine.

[19] Yang F, Chen R, Gu D (2020). Clinical Comparison of Full-Endoscopic and Microscopic Unilateral Laminotomy for Bilateral Decompression in the Treatment of Elderly Lumbar Spinal stenosis: A Retrospective Study with 12-Month Follow-Up. J Pain Res.

[20] Ko S, Oh T (2019). Comparison of bilateral decompression via unilateral laminotomy and conventional laminectomy for single-level degenerative lumbar spinal stenosis regarding low back pain, functional outcome, and quality of life - A Randomized Controlled, Prospective Trial. J Orthop Surg.

[21] McGrath LB, White-Dzuro GA, Hofstetter CP. Comparison of clinical outcomes following minimally invasive or lumbar endoscopic unilateral laminotomy for bilateral decompression. J Neurosurg Spine. Published online January 11, 2019:1–9. 10.3171/2018.9.SPINE18689 10.3171/2018.9.SPINE18689 30641853

[22] Ulrich NH, Burgstaller JM, Gravestock I, et al. Outcome of unilateral versus standard open midline approach for bilateral decompression in lumbar spinal stenosis: is “over the top” really better? A Swiss prospective multicenter cohort study. J Neurosurg Spine. Published online April 26, 2019:1–10. 10.3171/2019.2.SPINE181309 10.3171/2019.2.SPINE181309 31026821

[23] Mobbs RJ, Li J, Sivabalan P, Raley D, Rao PJ (2014). Outcomes after decompressive laminectomy for lumbar spinal stenosis: comparison between minimally invasive unilateral laminectomy for bilateral decompression and open laminectomy: clinical article. J Neurosurg Spine.

[24] Knio ZO, Schallmo MS, Hsu W (2019). Unilateral Laminotomy with Bilateral Decompression: A Case Series Studying One- and Two-Year Outcomes with Predictors of Minimal Clinical Improvement. World Neurosurg.

[25] Cavuşoğlu H, Kaya RA, Türkmenoglu ON, Tuncer C, Colak I, Aydin Y (2007). Midterm outcome after unilateral approach for bilateral decompression of lumbar spinal stenosis: 5-year prospective study. Eur Spine J Off Publ Eur Spine Soc Eur Spinal Deform Soc Eur Sect Cerv Spine Res Soc.

[26] Cavuşoğlu H, Türkmenoğlu O, Kaya RA (2007). Efficacy of unilateral laminectomy for bilateral decompression in lumbar spinal stenosis. Turk Neurosurg.

[27] Jayarao M, Chin LS (2010). Results after lumbar decompression with and without discectomy: comparison of the transspinous and conventional approaches. Neurosurg.

[28] Mariconda M, Fava R, Gatto A, Longo C, Milano C (2002). Unilateral laminectomy for bilateral decompression of lumbar spinal stenosis: a prospective comparative study with conservatively treated patients. J Spinal Disord Tech.

[29] Jitpakdee K, Liu Y, Heo DH, Kotheeranurak V, Suvithayasiri S, Kim JS (2023). Minimally invasive endoscopy in spine surgery: where are we now?. Eur Spine J.

[30] Armin SS, Holly LT, Khoo LT (2008). Minimally invasive decompression for lumbar stenosis and disc herniation. Neurosurg Focus.

